# Simulation and Modeling Efforts to Support Decision Making in Healthcare Supply Chain Management

**DOI:** 10.1155/2014/354246

**Published:** 2014-02-10

**Authors:** Eman AbuKhousa, Jameela Al-Jaroodi, Sanja Lazarova-Molnar, Nader Mohamed

**Affiliations:** ^1^College of Information Technology, UAE University, P.O. Box 15551, Al Ain, UAE; ^2^University of Pittsburgh, Pittsburgh, PA 15260, USA

## Abstract

Recently, most healthcare organizations focus their attention on reducing the cost of their supply chain management (SCM) by improving the decision making pertaining processes' efficiencies. The availability of products through healthcare SCM is often a matter of life or death to the patient; therefore, trial and error approaches are not an option in this environment. Simulation and modeling (SM) has been presented as an alternative approach for supply chain managers in healthcare organizations to test solutions and to support decision making processes associated with various SCM problems. This paper presents and analyzes past SM efforts to support decision making in healthcare SCM and identifies the key challenges associated with healthcare SCM modeling. We also present and discuss emerging technologies to meet these challenges.

## 1. Introduction

Advances in healthcare management systems helped improve the organizations operations and management. In particular, supply chain (SC) operations were vastly improved by the introduction of technological solutions. However, the overall enhancements still require more work to further improve operations, optimize performance, and minimize costs. Several models for SC and SC management (SCM) have been introduced in the healthcare business, yet the challenges involved slow down progress and hinder further benefit.

Simulation modeling (SM) is used in various fields to allow developers and users to represent a system and examine its operations using different possible scenarios and conditions. This allows developers to determine optimal operating conditions and also provide users with tools that allow them to explore various possibilities by changing procedures or conditions without actually disturbing the actual operational system. As a result, it is a logical move to introduce SM to the SCM in healthcare organizations. Since SCM in healthcare involves many parameters and requirements, it is essential for management and operational staff to be aware of possible results if the conditions or work parameters change. Users, given the right SM tools, can explore the best possible distribution/scheduling solutions for the available resources and find the best ways to satisfy the healthcare providers' needs.

Several research groups have approached this subject and many models and tools were created for specific types of SC requirements in the healthcare industry. The objective of this paper is to present and analyze past simulation and modeling (SM) efforts to support decision making in healthcare SCM, to identify the key challenges associated with SM in healthcare SCM, and to discuss new technologies emerged to meet these challenges.

The remainder of this paper is organized as follows. In Section 2 we provide the motivation and background. The particular design and management of healthcare SCM with examples of problems and challenges faced by the decision makers are demonstrated in Section 3. Recent SM approaches to support decision making in healthcare SCM are presented in Section 4 followed by a list of associated challenges in Section 5.  Section 6 discusses the new technologies to support SM and Section 7 concludes the paper.

## 2. Background and Related Work 

In this section we highlight the main motivation for this type of work and briefly explore the relevant concepts and work done in the field.

### 2.1. Motivation and Background

Competition within the healthcare industry is on the rise [1], and to stay in the market, healthcare organizations face the challenge to reduce operational costs while maintaining, if not improving, the quality of patient care and services provided [2–4]. In this scenario, supply chain management (SCM) becomes a high management concern as it costs as much as 40 percent of a typical hospital's operating budget, the second-largest expense for hospitals after labor [5]. Healthcare SCM is the process of delivering the right products in the right quantities to the right patient care locations and at the right time with satisfying service levels and minimized system-wide costs. By nature, SCM is a complex, dynamic, and distributed environment [6]. It is also governed by uncertainty and high variability [7]; this is so because it encompasses integrated and interrelated activities undertaken by different and distributed parties (i.e., suppliers, distributors, and consumers). Estimates of the potential benefit of an efficiently managed healthcare supply chain range from 2 percent to 12 percent of the hospital's operating costs [5, 8].

Efficient management of SC entails making informed decisions based on a holistic view of all elements that affect SCM process. To get there, SC managers in healthcare organizations need to have better visibility into their SCM processes to understand the causes of uncertainty and their impact on these processes. Managers need to be able to project the process performance and to adjust plans in real time in response to unexpected SCM events. They also need to investigate and validate solutions for different types of SCM problems without carrying out “trial and error experiments.” Not only because this approach is costly, but it is also extremely risky since the timely availability of products through healthcare SCM is often a matter of life or death to the patients. In this area, simulation and modeling (SM) has been presented as an alternative approach to assess solutions and to support decision making, risk management, and cost effectiveness analysis associated with various healthcare SCM problems [9, 10].

Simulation refers to imitating the operations and processes of a system in the real world; while modeling is the process of understanding and describing the behavior of a system [11]. The main purpose of applying SM technology is to analyze and evaluate a wide variety of “what-if” questions about a real-world system to predict its performance and outcomes after potential changes to the system. Thus, SM can serve as an analysis tool for predicting the effects of changes to existing systems or as a design tool to predict the performance of new systems under varying sets of input parameters or conditions [11, 12]. Accordingly, SM helps in reducing the cost, risks, and unnecessary human efforts if such experiments are experienced in reality. Moreover, participation in SM development allows concerned decision makers to develop deeper understanding of the problems they tackle and new perspectives about the relationship between the system's elements of interest and the measures of its performance [13, 14].

Many researchers [12, 14–20] discussed the role of SM in SCM and its potential to improve decision making in SC context. They presented the benefits of using SM in analyzing and evaluating SCs, process control, decision support, and proactive planning. They argued that SM is a very powerful tool for gaining insight into SCM. In view of these studies, the benefits of SM in SCM include: understanding overall SCM processes and characteristics; capturing SCM dynamics; modeling unexpected events and understanding their impact on SC; and minimizing the risk of changes in the planning of SCM process. Accordingly, it is important to have a good understanding of the benefits of SM in SCM optimization and what are the current issues and challenges in the field.

### 2.2. SM Tools to Support Decisions Making in SCM

Much research have been undertaken to develop SM tools to support decisions making in SCM. Here we briefly mention some examples of this work. Biswas and Narahari in [21] developed “DESSCOM”: a decision support for supply chains through object modeling, which enables strategic, tactical, and operational decision making in SCs. Ding et al. in [22] introduced a simulation and optimization tool “One” to support decision during assessment, design, and development of supply chain networks. Blackhurst et al. in [23] developed a decision support system, “PCDM,” for different decisions within the supply chain networks. In [24], the authors used SM technique to develop a decision support system to model manufacturing systems and to evaluate design alternatives. Wartha et al. in [25] developed a specialized and domain oriented decision support tool “DST-SC” that is easy to be used by nonexperts in simulation. DST-SC is also featured by its high degree of flexibility in modeling SC functions and its ability to handle large complex problems.

The application of SM in healthcare SCM is limited in comparison to other sectors, yet it is steadily on the rise [26]. Several studies [12, 13, 16] explored the value of SM to support decision making in healthcare SCM as in other industries. More studies developed SM tools to tackle problems in healthcare SCM. For example, authors in [27] addressed the problem of logistics and inventory replenishment through coordinating the procurement and distribution operations while respecting inventory capacities. Authors in [28, 29] dealt with the optimization problem of production and inventory management of blood supplies. Authors in [30] captured the relationship between vaccine supply and vaccine demand to calculate pediatric vaccine stock levels necessary for avoiding interruptions in vaccination schedules for children. Authors in [31] determined the optimal inventory policies for an inpatient hospital pharmacy with enhancement in cost performance. In [32], the authors developed a decision support system based on integer-programming models to address the problem of acquisition and allocation of medical materials. Lastly, authors in [9] developed a SM tool to analyze the supply chain of blood and blood products. They found that decision makers can use the knowledge created by SM to make better and less risky decisions regarding changes in SC. They concluded that SM can aid in increasing the overall quality of healthcare by allowing better allocation of scarce resources.

## 3. Healthcare SC Design and Management 

The complexity of the SCM problem magnifies with its focus on the healthcare industry. Healthcare SCM is designed to assure a high service level by maximizing the allocation of resources to respond effectively and promptly to the patient care needs. Beside that, it immediately impacts patients' lives rather than their livelihood. Therefore, healthcare SCM is different from SCM in other industries as it handles a diversity of items in widely varying quantities in response to the large number of diagnosis types and procedures. It is highly influenced by multifaceted legislations and by the central role of healthcare professionals [10]. While patients are the end consumers of products supplied through SC, they have no control on selecting these products. Unlike other industries, products cannot be promoted or auctioned when the expiry date approaches, and eventually they are destroyed. In addition, the constantly evolving technologies in the medical field result in short product life cycles and high costs for procuring healthcare professional preference items. Finally, it is difficult for planners in healthcare SCM to predict the frequency, duration, and diagnosis types for patient episodes and accordingly the associated product demands [8].

In healthcare, SCM “enables” patient care through supplying the diverse medical professionals with products and services they need to deliver prompt and best quality medical care. In addition, there are many consumers (patients and medical professionals) with a high variety of needs. How those needs are satisfied becomes another challenge and each consumer's unique request must be addressed. At the same time, SCM is set to “enable” the strategy of healthcare organizations maximize patient care and minimize cost. This can be achieved by ensuring product availability, minimizing storage space (to maximize patient care space), reducing material handling time and cost, and minimizing nonliquid assets (inventory) [33]. Thereby, decision making processes in healthcare SCM must consider many elements such as cost, profitability, standardization, and inventory management.

**Figure 1:**
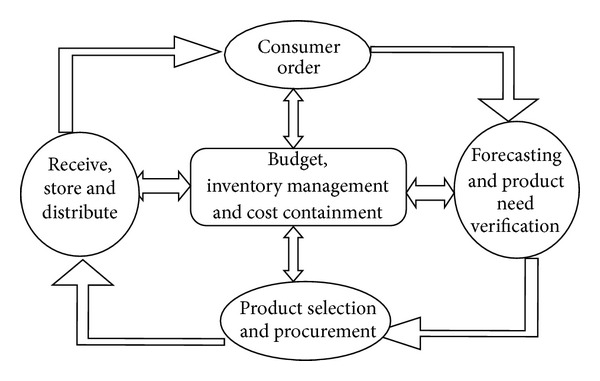
Modern Healthcare SCM Process.

The modern SCM process in healthcare (see Figure 1) is divided into a series of cycles each perform at the interface among various successive stages.

- Customer order is triggered when a product's level reaches a certain low level as it is consumed through usage and sales.
- Forecasting and product need verification verify the need to order new stock based on usage and sales in addition to studies of trends, product availability, on hand stock and product cost.
- Product selection and procurement: used to select the appropriate product to order based on availability, cost verification, order quantity, lead time and delivery date.
- Receive, store, and distribute: used to receive ordered products based on approved orders, also to verify that the products are delivered in the right quantity, at the right price and on time.
- Budget, inventory management, and cost containment: This represents the SC fiscal responsibility to the organization as general policies and budget concerns are addressed and orders are optimized to meet overall organizational goals.

The process illustrated in Figure 1 is a typical SC process. However, instead of the consumer being the central focus, many organizations are looking at the cost to revenue approach as the central focus. By placing this process focus into perspective, successful SC managers find their competitive advantage by optimizing the balance between the concept of meeting consumers' demands and the fiscal responsibility. Thus, effective management of healthcare SC is driven by performance which measures represent this balanced approach which includes: total cycle time; product availability; quality; responsiveness; compatibility with policies and guidelines; flexibility; and cost effectiveness [34]. Decisions taken to achieve SCM goals within high performance measures are classified at strategic, tactical, and operational levels. Strategic (long term) decisions include decisions concerned with SC structure. Planning (medium term) bridges the gap between strategic decisions and operational decisions concerned with the day-to-day functions [35].

*Strategic Decision Making.* In the SCM process, the price elasticity of demand plays an important role. In healthcare SC there are many products available to the end user that perform the same or similar functions. The challenge becomes ordering the best product at the best possible price that satisfies the needs of the majority. This becomes critical when budgetary restrictions are in place. Within the healthcare industry as in all other industries, inventory is viewed as assets. SCM's goals are to balance cost with the right amount of inventory to sustain operational workflow. It is a difficult decision to make when dealing with an organization that has many consumers (healthcare professionals) that have their specific product preferences for their own individual reasons. Standardizing products that meet the needs of all involved within optimized inventory levels is a tough decision for SC managers to make. Deciding the location and capabilities of warehousing facilities is another challenge in this area.

*Planning Decision Making.* To balance consumer needs and the organization's profitability goals, the consumers' input becomes valuable at the time the organization needs to decide how that will fit into meeting financial goals. As shown in Figure 1, the consumer is taken out of the center and replaced with three necessary elements that are the foundation for planning today's healthcare SC and for customers/suppliers' relationships. Identifying where profitability and sustainability will occur make it easier for SC to focus on the products necessary to provide the needed services. Budgetary limitations lead healthcare SC managers to incorporate new functions and policies into their procurement, inventory managements and distribution cycles.

*Operational Decision Making.* One of the biggest challenges facing healthcare SCM operationally is maintaining sufficient inventory levels to sustain quality and timely patient care. The other is wastage (i.e., too much inventory which often leads to a high product expiry rates). There are a number of factors that healthcare organizations are confronted with: contributing to wastage due to poor planning, not understanding appropriate inventory par (min/max) levels, and not monitoring budgetary guidelines. In an effort to keep the consumer happy SC departments need to keep plenty of everything. However, too much of a good thing can create a snowball effect and end up costing the organization heavily. Inventory is often viewed as a potential source for revenue. Having an overstock (wastage) of inventory adds to organizations opportunity costs; money that would have otherwise been spent elsewhere within the organization. In the healthcare SC environment, a considerable amount of inventory is moved on a daily basis, it is then necessary to maintain appropriate stock levels of those items, many of which are quite costly. Operationally, the challenge is in finding and then maintaining inventory balance so that hospital budgetary requirements and consumer demands are met.

## 4. Proposed Simulation Models 

Simulation models facilitate the design and management of healthcare SC through producing a holistic view of all involved elements and providing “what-if” analysis tools. With respect to SC design issues, SM can support decisions concerning process flows, localization (location of facilities, distribution systems), selection (suppliers, partners, products), and size (capacity of facilities). With respect to SCM issues, SM can support decisions concerned with policies, planning processes, inventory management, and suppliers/consumers collaboration agreements. Here we present a significant number of proposed modeling efforts to support decision making processes in healthcare SCM. We analyzed these efforts according to the key components of SCM [36]: scope, problem, decision variables, objective, monetary value, customer service initiatives, and constraints. Decision variables aim to limit the range of decision objectives in SC and to measure its performance according to these objectives. The monetary value reflects the cost efficiency and profitability of SCM activities while the customer service initiatives include the two main elements for consumer satisfaction: product availability and response time. Constraints represent restrictions placed on SC, which are generally pertaining capacity, service compliance, and the balance between demand and consumption. Assumptions made by the modelers during model development to simplify the reality of SC are also addressed in our discussion.

### 4.1. Optimizing Scarce Drug Allocation

Swaminathan in [37] proposed a SM-based decision support system (DSS) to support the Drug Distribution Project (DDP) in the state of California, USA. DDP aimed to manage fair and equitable scarce drugs (those with demand greater than supply) distribution in the state with the involvement of 150 clinics and 25 pharmaceutical companies involving 125 drugs in 20 drug categories. The allocation problem as described was nonlinear, multiobjective, and large in size. Therefore, the proposed DSS utilized a multiobjective optimization model and a heuristic solution to accomplish optimized distribution while taking into account efficiency, effectiveness, and equity of the drug-allocation process.

Allocation efficiency in the model was measured by the extent to which all drugs are distributed to clinics with a maximum dollar value of drugs allocated (equal to a minimum left over budget) in a given ordering period. Effectiveness was measured by the extent to which every clinic received the drugs it needed. The model uses a weight matrix (*π*_*ik*_) to determine the importance of drug *k* for clinic *i*. To achieve equity, the model uses an allocation heuristic approach to get each clinic a fraction that is weighted by *π*_*ik*_ and proportional to the ordered amount of any short-supply drug. Relevant decision variables are defined to be the dollar value of drug *k* received by a clinic and the binary value (0 or 1) that represents whether clinic *i* got any allocation of drug *k*.

Given these performance measures, the model defines two objective functions: (1) minimize the leftover budget in any given period (to achieve efficiency) and (2) minimize the difference between allocation ratios and weighted orders from clinics (to achieve effectiveness and equity). The model was set to perform according to several constraints as follows.

- Clinic constraints: clinics should not exceed their allocated budget in ordering drugs.
- Pharmaceutical firm constraints: dollar value of total disbursement should not exceed the limits in the settlement agreement.
- Allocation constraints: dollar value of allocated drug to a clinic is less than or equal to the ordered amount and meets at least the minimum order quantity set by the clinic for each drug.

The model solution was developed to first identify scarce drugs and then find a fair allocation among clinics considering all constraints. The performance of the allocation heuristics depends greatly on the priority provided by the decision makers through the weight matrix (*π*_*ik*_).

The proposed DSS in this work was proven successful at providing efficient, effective, and fair methods to allocate scarce drugs. However, the primary assumptions did not consider procurement centers for different clinics in one region. Orders from such centers tend to overshadow other clinics. Although the model was adjusted by normalizing the base weights for all large clinics, there is still an issue with the model scalability and complexity. The large size of the weight matrix (*π*_*ik*_) increases the complexity of priority weight determination. Finally, this model is specific to DDP with no immediate applicability elsewhere.

### 4.2. Optimizing Drug Inventory

Ana et al. in [31] presented another modeling approach for inventory and ordering policies for drugs for inpatient hospital pharmacies. The objective was to minimize wastage and holding cost while maximizing prompt access to the drugs. The approach used patients' medical condition information to determine the appropriate inventory level of raw materials and finished pharmaceutical drugs. Markov decision process (MDP) was used to model the drug demands as a function of patient condition and accordingly to decide the appropriate level of drug inventory and the drug order quantities. The objective functions were to (1) minimize all associated costs including stock-out cost for both finished and raw goods and inventory expiry cost and (2) maximize timely access to the required drugs. The main assumption was that there was no back-logging of demand. The patient demand was assumed to be fulfilled at the same day even if it involves procuring the drug from a different hospital. The model also assumed that out-of-stock goods are received immediately after order placement, while raw materials have one period delay. State definition in the corresponding MDP involved two components.

- Patients: two types of patients are modeled by two distributions of corresponding mean values to reflect high and low demand variability. A third type of patients is defined to represent patient discharge (absorbing state). For example, a patient with severe condition will be of type 1 with a higher mean of demands. The unique patient demands (Q) were modeled as discrete nonnegative values based on stationary probability mass function. The maximum demand was assumed to be a finite number. The demand's mean decreases from patient type 1 to 3.
- Inventory is defined as a multidimensional vector (I) to meet the assumption of two drug forms: raw and finished.

The model also defined a set of transition probabilities to capture changes that may occur in patient classification from one type to another, as well as changes in demand. Arrival rates were also defined by patient's type. At a given period of time, system state (I, Q) was obtained to support evaluating the decision of how much drug quantity and raw material units to order based on the expected patients' demands. The model was solved numerically using backward recursion to determine the optimal inventory policy. Results indicated that the optimal policy has a base stock structure within which base stock levels are dependent on the raw and finished goods as well as the patient types mix. Accordingly, two policies were developed and compared: adaptive policy (AP) that is based on the MDP solution and fixed policy (FP) that uses a fixed base stock level. In the defined multiscenario experiment set, AP outperformed FP.

To this extent, the model overlooked the production cost that may concern some inpatient pharmacy environments. Healthcare SCM have no control over production costs; however, understanding the initial cost of products can help when negotiating effective pricing. Moreover, it is unrealistic to tie the patient type to the demand variability. Patients may be discharged with higher number of drugs than needed in the ward. More concerns are related to the worldwide shortage of the drug that may impact order quantity, sudden increase in demands, and the drug availability from other facilities. Fixed one day delay window for the raw materials delivery is also a strong assumption to make; as it requires to learn how much stock a supplier usually keeps on hand to ensure these materials availability.

### 4.3. Optimizing All Products Inventory

Still in the area of inventory optimization but moving towards a more general solution; Little and Coughlan in [38] developed a constraint programming optimization model to determine the optimal stock levels of overall products in hospitals controlled by space, delivery, and criticality of the products. The proposed system is aimed to meet the requirements of healthcare SCM. These include achieving high service levels with least delivery cost; ensuring that materials are not overstocked or becoming expired; supplying all needed products with no delay or no out-of-stock problems; and reducing the cost of stock hold and distribution.

Thus, the model defined three decision variables that were associated with each product to be stored: (1) service level (initially set between 90% and 99%); (2) frequency of delivery (initially set between daily to once every ten days); and (3) stock-up amount (a positive amount). The objective functions were set to (1) maximize the minimum service level and (2) maximize the average service level. The model approached the problem as a type of “unbounded knapsack problem” with the knapsack being the stock hold (available space) in this case. The solution for this problem involved determining a maximum value to be placed in the knapsack within the weight constraint. The weight constraint corresponds to the inventory volume to control the amount of each product to be stored. With the assumptions that the products demand is normally distributed, the constraints set for this model include the following.

- Inventory constraint: to ensure that the relationship between the decision variables is kept consistent.
- Space constraint: to ensure that the volume of all products of different types to be stocked up is less than the maximum available space.
- Criticality constraint: to allow consumers to impose constraints to fix any product to the highest level of 99%.

The model was implemented using optimization programming language (OPL) to generate an optimal inventory policy to achieve a solution that has many products of high importance at high service level. To find such solution, the model adopted three main search strategies: (1) select the products by highest importance; (2) select products in order of increasing size; and (3) select products by decreasing demands. The model was experimented within a real time setting (Intensive Care Unit at Cork University Hospital, Ireland) in two different sets. The first set of experiments was intended to track how varying the decision variables results in various optimal policies; while the second set was intended to explore how different objective functions and search strategies improve the quality of the inventory policy. For these experiments, the model was able to quantify and predict the inventory policy and how it behaves in response to the changes in space and delivery pattern.

This proposed work focuses on the issue of limited product storage space in hospital sites under the assumption that products are supplied with regular (normally distributed) demands. However, products demand in hospitals usually exhibits highly dynamic and uncertain patterns. Any model to determine the optimal stock level in hospitals should realize (or predict) changes to demands and take action to adjust policies or supplies accordingly.

### 4.4. Optimizing Sterilization Logistics

In a different direction in SCM design, van de Klundert et al. in [39] deal with the logistic process optimization problem in SCM by presenting a potential model to optimize logistics or flow of sterile instruments. Flow of sterilized items takes place between the central sterilization service department (CSSD) and the operation theatres (OT) in hospitals. In practice, the demand and consumption of sterilized items are determined by the number of planned and emergency surgeries. Sterile instruments are consumed in nets rather than individual items. Each sterile net typically includes a group of items needed for a particular surgery. An effective logistic control principle applies to replenish (stock-up) immediately all items (nets) to the sterile storage area in OT and to process used items and return them to the storage area before the end of the day. This process takes more than half a day in spite of the fact that the CSSD is often located near the OT. However, this principle is insufficient as it first requires maximum storage capacity in a critical area such as the OT where the space is more valuable for patient care; second it involves extra working hours by CSSD for items that may not be needed by the next day; and lastly it adds unnecessary transportation cost.

Suggested model optimizes the logistic process by changing the aforedescribed principle and redesigning the process to improve item availability and reduce cost at the same time. It first assumed outsourcing CSSD and that sterile nets are used only once per day. After that, it was suggested that the replenishment process is completed on a weekly basis with three costs involved: transportation cost, OT storage cost, and instrument usage cost. The proposed model addresses two problem formulation settings.

- Deterministic optimization: assuming that all surgeries and resulting sterile instrument net usage are predictable, in this case, sterile items can be delivered just in time before a surgery begins. The objective function for this model requires minimizing the total cost by minimizing the number of transportation delivery for a given OT schedule considering the storage capacity at the OT. To achieve this, the model was set to select a set of delivery moments for sterile nets that serve the largest number of surgeries scheduled in blocks. The underlying assumption was that sterile nets were used in the block directly after delivery; that is, they did not need storage.
- Nondeterministic optimization: this approach disposes the assumption that the OT schedule is predictable and solves the problem with the assumption that the number of required nets is unknown. Basically, the only way to deal with unplanned usage of sterile nets is by keeping and replenishing a safety sock. Accordingly, the system proposed four strategies.
  1. Arrange for sufficient safety stock in advance. This strategy deals with stochastic demands in a static manner and does not require information exchange on unexpected use.
  2. Include both planned usage as well as safety stock in the original planning. This strategy uses prior knowledge and does not require information exchange on expected usage.
  3. Schedule delivery only for planned usage; and guard against unplanned usage by an initial safety stock. When stock level drops below the safety stock level, the transportation plan is dynamically reoptimized based on real time information to include replenishment of nets which are below the safety stock level.
  4. Schedule delivery for both planned and expected usages; and guard against unplanned usage with an initial safety stock. The transportation plan is dynamically re-optimized based on real time information to include replenishment of nets which go below safety stock levels. This strategy depends on prior knowledge and real time information.

The optimal delivery schedule forecast in all cases is computed using a dynamic programming approach. Proposed strategic solutions were compared against reference cost in a simulation environment. Policies with stochastic demands resulted in lower cost than the reference cost. The practical and theoretical work presented in this work shows that up to 20% cost reduction is possible through the optimization of the flow of sterile instruments between sterilization departments and hospital OTs along with processes' streamlining and materials' standardization. However, the transportation cost increases when outsourcing CSSD, which is balanced by the OT storage cost decrease. This would happen only if it is not possible to increase the storage capacity of the OT to handle weekly supplies of nets. In turn, this requires considering alternative solutions such as cheaper remote CSSD, cheaper intermediate storage areas near the OTs, or other solutions to counterbalance the increase in transportation cost.

### 4.5. Optimizing SCM Logistics

Rego and Sousa in [10] also dealt with logistic process optimization, however with redesigning the whole configuration of the hospital SCM. The main problem of current hospital SC configuration lies in the lack of coordination between the strategic decision level and the operational decision level. This increases the challenges for the decision making processes in whether, when, and how to use the existing SC structure, for example, deciding where to locate storage centers, how to use these locations for existing facilities, how to move materials between centers, and how to schedule people to cover these centers. The goal of the proposed model was to improve the logistics organization of SCM services in the hospital to enhance service quality and reduce overall cost, response time, and storage space. The model was developed based on Graph Theory [40] to describe multistage, multilevel and multiproduct production and distribution planning system. The model represented the multiperiod dimension of the problem through replication of SC with “inventory edges” connecting storage areas in subsequent periods. Decision variables associate the quantities supplied to the supply path (edge). Thus, sending a given inventory through one edge depends on the supply path that the quantity traveled before. The objective function is to minimize the total cost, which includes acquisition, transportation, administrative, and inventory carrying costs. The model assumed that administrative cost is fixed and that storage constraint is constant throughout the planning process. The model was set to perform under the following constraints:

- demand satisfaction (no stock-out allowed),
- flow persistence between SC members (represented by nodes in the graph),
- storage capacity,
- aggregating all items,
- producers supply capacity affecting all potential buyers,
- non-negativity and integrality of decision variables.

The solution of the model involved selecting traveling paths with the minimum cost to distribute inventory. Given the combinatorial nature of the addressed problem and the size of instances involved, the approach developed to solve this model which adopted a hybrid algorithm based on metaheuristic technique, Tabu Search (TS), and Variable Neighborhood Search (VNS). The solution suggested three-neighborhood structure (NS) for path completion or path substitution while delivering a certain quantity.

- Select paths with minimum cost, ignoring the current solution structure (other paths of current solution may use common edges of the underanalysis path).
- Select paths with minimum cost considering the current solution structure.
- Select a new path by randomly choosing the chain elements while satisfying the capacity constraints.

Demand was described using normal distribution to represent units of high and low demand. The obtained solutions returned different cost values according to the NS used. It is then left for SC mangers to assess how reasonable these solutions are before selecting the best one.

The preliminary computational results of the model showed the potential of the approach in solving large scale and diversified SC configuration problems. Although the approach does not consider sudden increases in the demand, it may be incorporated in a DSS to simulate, discuss, and negotiate SC coordination partnerships between neighboring hospitals and other members such as suppliers. In addition, the flexibility of the proposed approach allows its application to SCs with various topologies and uncommon cost characteristics. However, all of these potentials are still to be evaluated with real data obtained from a real hospital SC.

### 4.6. Optimizing Logistics Activities

Unlike the last two models, Lapierre and Ruiz [27] addressed the problem of logistics optimization based on a schedule-oriented rather than an inventory-oriented approach. An inventory-oriented approach focuses on assuring sufficient inventory levels but does not account for human resources activities. However, SC managers in healthcare require answers to many questions beyond inventory control such as those related to the planning and control or scheduling of activities and manpower resources. Examples of these questions are when each employee should work? How often and when to replenish/visit each care unit (CU)? How often and when to call suppliers? In addition, in inventory-oriented approach all decisions are based on cost or service levels and do not account for other beneficial aspects attached to activities' control. Manpower resources in the hospital SCM are required to accomplish four main activities: (1) procurement and purchasing, (2) reception and handling, (3) replenishment preparation, and (4) distribution and inventory control at CUs. The proposed model aimed to support the optimization of the SCM through presenting a solution to schedule and coordinate these activities while respecting inventory cost and capacities. The presented solution is based on two modeling approaches that account for the many scheduling decisions concerning the SC managers.

The proposed model was designed to decide: (1) when each CU will be visited and which products will be delivered in each visit; (2) when each supplier will deliver to the hospital, and which products are included in each delivery; and (3) what inventory quantity is shipped directly to CU on the same reception day. Direct shipments to CUs require less delivery time, but it can consume more time as it may lead to more frequent purchases. Thus, mainly three decision variables were identified with respect to the service sequence at a given period: suppliers' delivery, CU visits, and manpower time. To simplify the problem, the assumption is made that only three large suppliers can visit the hospital several times a week and that there is a total of 43 products to be delivered to 23 CUs by the three suppliers. For the testing purposes, only datasets of 10 and 20 products were used and the storage capacities of CUs were reduced accordingly.

The model included two types. The first type (M1) was an inventory cost-oriented model with an objective function to minimize the total cost of inventory and human resources. The model associated a utility function (stock value) to each product which accounts for their price, volume, weight, and variance of demands. By minimizing this stock value function, the model maximizes visits to the CUs. The second type (M2) aimed to provide activities' schedule that balance the workload over the week days and introduced this objective into M1. The objective function of M2 is to minimize the total manpower use time for all SCM personnel at the time of any given activity. Both models, M1 and M2, are set to perform under several constraints as follows:

- set time restrictions on manpower to accomplish the required activities;
- direct deliveries are restricted to products received within the current period;
- demand satisfaction;
- respect of storage capacity (weight and volume);
- no product is received if the supplier does not visit the hospital;
- products at CU can only be replenished if unit is visited.

Outputs of M1 and M2 produce significant information on SCM personnel's schedules and amount of manpower hours per day, distributed among the activities of SCM. As in the previous models, the heuristic techniques, TS, and VNS were applied to solve M1 and M2. The quality of the obtained solutions was compared considering three criteria: (1) carrying cost; (2) uniform workload distribution; and (3) required working time. The best solutions suggest spending more time in procurement and inventory control operations than the current situation in hospitals. This means that hospitals should order more frequently and reduce stock levels in central stores. The model also suggested controlling inventory levels in CUs by dedicating a person there to make replenishment decisions.

While the approach may help SC managers in hospitals to improve logistics by better coordinating their procurement and purchasing activities, the information it produces may be used only to fix a schedule for the required number of workers to accomplish everyday activities but not the details of this schedule. Examples of schedule details include the special skills of these workers, delivery paths, and the assignment of different activities to different workers. The different paths may impact the delivery time while worker activity assignment is controlled by the execution time of each activity, priority relationships between tasks, and break periods. In addition, while the approach succeeded in performing “what-if” analysis to compare different strategies, it failed to provide a tight schedule as an optimal solution. Lastly, the approach is computationally expensive. In the experiment setting, the naïve assumption was made for small number of products, suppliers, and CUs.

The summary data on these simulation models is in Table 1. It is notable that all models approached the optimization problem at different levels of SCM. In healthcare SCM, optimization addresses the general problem of delivering products to consumers at the lowest total cost and highest level of service. It is also worthy to highlight that only two models among the above models (Sections 4.1 and 4.3) reported real time evidence of implementation and testing. The implementation challenge is discussed in the next section along with the rest of the challenges and concerns that prevent a wider use of SM in solving healthcare SCM problems.

## 5. Challenges for SM in Healthcare SCM 

The increased interest in SM for healthcare SCM is not without its challenges. In the light of models discussed above, the most challenges faced by simulation and modeling community in healthcare SCM are as follows.

*Collecting Sufficient Amount of Related Input Data.* A third of the time in SM projects is devoted to data gathering and validation. Yet a lot of money is wasted in many SM projects due to solving the wrong problem as a result of insufficient or irrelevant input data [41]. For all the approaches discussed above, SM builders collected data from the manually entered historical records of SCM or via interviewing SC managers and employees. While this process is important to develop a better understanding of the problem, it is time-consuming and subject to overwhelming and unnecessary details. Also, the manually entered data about the status of inventory in a SC is seldom accurate.

*SM Validation and Verification.* Validation and verification aim to determine the accuracy of the model and the SM project by finding errors and correcting them. These are fundamental yet very time-consuming activities [42].

*Implementation.* A large proportion of SM studies in healthcare demonstrate a conceptual level and only few report evidence of implementation [43]. A recent study in 2011 by Katsaliaki and Mustafee [16] surveyed 201 healthcare SM related research studies but found only 11 which reported the implementation of results to healthcare organizations. Our study investigates currently adopted or potentially eligible for adoption in the healthcare industry thus offers a different view of the available features and the shortcomings of these models. In addition, this allows for identifying the challenges and issues that need to be addressed to reach better and more effective models. Most of the developed models in academic settings are not widely accepted by healthcare organizations. This may be attributed to several reasons, amongst the top, are implementation cost and the issue of model generalization.

*Implementation Cost.* The implementation of SM can arrive at a significant cost to the healthcare organizations, in particular for the medium and small sized entities. SM projects may require expensive information communication and technology (ICT) infrastructure while the resources in healthcare settings are scarce and most preferred to be allocated to improve clinical services.

*Generalization.* Most of development models are specific to the hospitals that they were developed for and cannot be immediately applied in other hospitals [44]. Hence, there is imperative need for generic models with a high degree of flexibility and scalability.

*Growth in Models' Size and Complexity.* Models for healthcare SCM are growing in size and complexity due to the increasing number of objects and events in healthcare SCM, and the shift towards partnerships between different hospitals and other SC members. This leads to a long run time problem in modeling and execution speed. However, decision makers are in need for a tool that can provide immediate solutions rather than when the answer is already out of date.

*Representation of Human Decision Making.* Most, if not all, proposed SM for healthcare SCM represent SC entities and focus on modeling resources scheduling and allocation and work processes rather than the representation of complex decision making processes made by SC managers [45].

*Match between SM Techniques and SCM Problems.* When selecting the SM techniques to tackle a SCM problem, it is crucial to choose the most appropriate technique to ensure an accurate representation of the problem [46]. In practice, however, it appears that the selection of which SM technique to apply regardless of the problem is the one the modeler knows best and more familiar with [46, 47]. Towards helping in this end, authors in [48, 49] propose an approach to develop a framework to assist practitioners in selecting the appropriate technique for SCM challenges.

*Expert Modelers.* The expert modelers are few and far in between [17]. Thus, model construction, in most cases, is left to insufficiently trained SC managers and analysts. Yet, constructing good descriptive or optimization models requires huge efforts, experience, and expenses that are, sometimes, more than what an expert modeler can accomplish or more than what a company is willing to invest [41].

*Expert Users.* SM requires users to be familiar with software and statistics knowledge. However, most of SC managers and analysts are nonexpert SM users. Thus, SM software should be easy to learn with an easy-to-use graphical user interface that helps users in problem definition, design of computer experiments, simulation runs, access ready information, and results analysis. Results should be presented in understandable and interpretable format with the ability to transfer these results to be used in different reporting tools.

*Integration of Existing Models.* The integration of existing models is an issue of two levels.

1. Intermodels integration: as we have seen in Section 3, proposed models perform at different levels of SCM. The advances in integrating these models will have value in saving extra model building efforts; exchanging information between SC members; and reducing overall execution time.
2. Models legacy system integration: most developed simulation models are independent and standalone tools. Advancements in integrating these with HC legacy systems such as inventory management systems or electronic resources planning (ERP) are critical to address the issue of reluctance of sharing information among different SCM members and other decision making entities in hospitals as well as the issue of the lack of and inaccurate input data [14].

## 6. Promising Technologies to Support SM for Healthcare SCM

To fully exploit the SM opportunities in healthcare SCM, several and different technologies have been tried to meet the challenges presented above. Some of the following technologies may seem irrelevant to SCM in healthcare, yet the popularity and usability advantages offer a potential shift in paradigms that will incorporate SCM with some of them to leverage some potential problems. Here we briefly discuss some of the most promising technologies.

### 6.1. Agent-Based Simulation (ABS)

ABS is a relatively new paradigm that is based on the concepts of multiagent systems (MAS) and robotics from the field of artificial intelligence (AI) [50]. ABS represents a complex system by a collection of agents programmed to follow some behavior rules. The agents are “objects with attitudes” [51] that are designed to mimic the behavior of their counterparts in the real word. Unlike objects and entities in traditional simulation techniques, agents are capable of making independent decisions and showing active and social behaviors. ABS uses a bottom-up modeling approach within which the behavior of agents is defined at the individual level and then the system properties emerge from its agents interactions. ABS is attracting a great deal of attention because it helps understand the increasing complexity of real world by providing a natural representation of the system and by producing the unpredictable behavior of a group of people according to their independent decisions (emergent phenomena) [52]. The characteristics of ABS make it applicable to simulate problems ranging from the strategic level to the operational level; however it is more suitable for strategic problems [48, 53] where human behavior, information sharing, and collaboration are involved. Nevertheless, ABS is not widely used in industry due to the lack of commercial software and the fact that ABS is computationally intensive [46, 53].

### 6.2. Radio Frequency Identification (RFID)

Radio Frequency Identification (RFID) has been identified as “*one of the ten greatest contributory technologies of the 21st century*” [54]. RFID is used to track physical moving or fixed items and to collect desirable data from these items. RFID consists of (1) tags (small integrated circuits) that store information and can be attached to the items; (2) a wireless network of electronics “interrogators” that reads the information on the tags; and (3) a middleware that bridges the RFID hardware with enterprise applications. Electromagnetic waves are used for sending and receiving information between tags and readers [55]. The introduction of RFID technology brought countless benefits for SCM such as improving the speed and accuracy of tracking inventories; reducing inventory levels; reducing operating costs, and improving the efficiency of work process [55]. Tajima in [56] described 15 different benefits for using RFID in SCM. Amini et al. [57] explored one extra benefit of using RFID as the data source for SM development. They demonstrated that with the RFID capability to allow selective data collection and organization, it enhances data availability, at various levels of detail and complexity defined by the user.

Accordingly, RFID contributes to mitigate the aforementioned challenges associated with the traditional forms of data collection in SM projects. This was followed by several studies that used SM techniques to solve problems with RFID-enabled SC systems. Most of these studies are reviewed by Mehrjerdi [58]. RFID technology is developing; however, it promises a good opportunity for improving the accuracy and efficiency of SM for healthcare SCM.

### 6.3. Distributed Simulation (DS)

**Figure 2:**
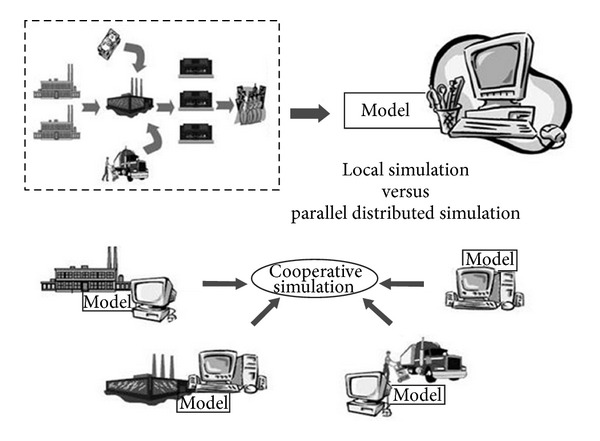
The two paradigms for SCM simulation [62].

Distributed simulation (DS) depends on the distributed systems technology to enable the execution of a single run of simulation program across multiple interconnected processors [59]. The application of DS within the context of SCM in general is motivated by the distributed physical environment of SC and the need for information exchange between its also distributed parts and/or members. DS contributes to not only reducing the simulation execution time but also to integrating the different models that already exist [60]. Thus, a simulation model of SC can be designed traditionally as a standalone single model run on one computer (local simulation) or using multiple integrated models representing the different parts of the SC that run in parallel on multiple synchronized computers (parallel distributed simulation). PDS allows for designing and realizing complex SCM simulation systems that cross hospital boundaries to a wide range of suppliers and consumers. Within PDS-based systems, the different models representing each entity are self-contained with the ability to share the common information as needed [61].  Figure 2 as presented by Iannone et al. in [62] illustrates the two paradigms.

The potential of DS for healthcare SCM has been explored in various studies. For example, Katsaliaki and Moustafee in [13] compared the execution time of the standalone healthcare SC simulation with its distributed counterpart. They found that the run time of the standalone simulation increases exponentially as the size and complexity of the model increases while using DS decreases the execution time for large and complex models. Mustafee et al. in [63] investigated if using DS can speed up the traditional simulations for blood SC in UK. The results indicated that DS achieved better performance as the model grows compared to the standalone simulation.

One particular challenge for DS lies in properly managing the communication between the distributed computing models or nodes. There are two frameworks suggested to handle this challenge [62].

- Network structure: based on distributed protocols to facilitate the interaction among point-to-point interconnected nodes and to update simulation states.
- Centralized structure: based on a centric instrument (message broker) or software (middleware) to manage communication between the simulation nodes.

However, the first framework suffers from the problems associated with traditional point-to-point networks which include no support for routing logic and limited support for heterogeneity; and complexity increases as more nodes are added to the simulation. This is supported by the results in [63] where the execution time of distributed simulations increased exponentially as the number of hospitals (nodes) increased. The second framework, on the other hand, supports flexibility and scalability by separating the communication activities from the model's activities using a message broker. The message broker takes the responsibility to filter, process, and distribute messages as needed between nodes. It uses the node's identity, message type, or message content to perform a logic routing while managing communication between the nodes. It also provides adaptors to generate uniform data formats. Hence, this framework is becoming widely used to develop the parallel distributed simulation (PDS).

### 6.4. Service-Oriented Architecture (SOA)

Service-oriented architecture (SOA) is an architecture that leverages open standards to represent software or system functions as services through well-defined and stable interfaces specified with a service contract [64]. A service only exposes its interface on the web while the service's contract specifies the purpose, functionality, constraints, and usage of this service. SOA is driven by the emergence of Web Services which became the preferred method to build SOA environments. SOA allows developers to create their applications using the services provided by different organizations and published on the web. The perceived benefits of SOA include supporting on-demand business, improving information sharing, lowering systems complexity, reducing integration cost, and improving efficiency [65]. SOA also increases flexibility in responding to dynamic changes in the application requirements and performance [66].

In the area of SM development, SOA contributes to mitigate the concerns related to the long development time, integration with legacy systems, implementation costs, and shortage of expert modelers. Simply, it provides on-demand simulation services for designers to construct good descriptive SCM models in a short time and lower cost. In addition, adopting SOA in developing simulation models enables designers to adjust models in a flexible and cost-effective manner. Several articles presented SOA frameworks for easy development of SM and distributed simulations such as SIMPROCESS [67], SOAr-DSGrid [68], and DDSOS [69].

### 6.5. Cloud Computing (CC)

Cloud Computing (CC) is defined as “*a method of running application software and storing related data in central computer systems and providing customers or other users access to them through the Internet*” [70]. By definition, CC allows organizations to shift the burden of applications development or the whole ICT infrastructure implementation and management to a third party: the cloud service provider (CSP). The CSP responds to the organizations' needs of outsourcing their ICT by offering flexible and scalable service architectures and through “pay as you use” contracts [71]. CC can be seen as an evolved model of DS and SOA technologies. The CSP in the cloud provides three major services [72].

- Software as a service (SaaS) provides different services and applications for clients to use over the Internet.
- Platform as a service (PaaS) provides platforms or run-time environments to clients. It offers a wide variety of resources like databases and development environments with basic services to build and deploy clients' applications.
- Infrastructure as a service (IaaS) provides infrastructure resources and allows for remote storage and applications' execution. At this level, the CSP takes care of the daily procedures of using and maintaining systems in the cloud.

The advantages of CC include reduced cost of ICT; flexible payment models such as pay as you go or pay per service; highly reliable/available services and resources; up-to-date tools to facilitate applications development; remote and location-independent access; reduced ICT management responsibility; ability to handle unexpected higher or lower demands for resources (scale up or scale down); ability to share resources and costs across a large pool of clients and offers security mechanisms [73]. CC solutions can support the simulation community by the following:

- providing the computing platform and infrastructure for model builders to develop their models and/or to execute simulations and get results without the cost of ownership;
- providing the simulation software in a SaaS manner in which every software function is treated as service.

Beside saving time and efforts consumed by the software development process, Simulation Software-as-a-Service, SimSaaS, provides the advantages of scalability and the multitenancy architecture (MTA). MTA [74] maximizes sharing of software, data and data schemas by multiple tenants/partners. Using MTA, SimSaaS provides the simulation system with the ability to add/remove/modify partners; address the partners' accessibility controls; distinguish partners' simulation interaction message during executions; and isolate partners' own specific data. In comparison to the DS technology discussed earlier, SimSaaS presents promising potentials to meet most of the challenges for SM of healthcare SCM. Several recent studies explored in detail how SM can benefit from CC. Other studies propose solutions for SM in the cloud. For an example, Tsai et al. in [75] propose SimSaaS with a MTA configuration model and a cloud-based runtime to support fast and scalable simulation development to be run in a flexible cloud environment. Guo et al. in [76] present a SimSaaS architecture to support automatic deployment of simulation services to run experiments defined by clients.

Among aforementioned technologies, RFID and DS are still the only two practically tried technologies to aid SM for SCM in general and in the healthcare context in particular. The rest are still at the conceptual level. This implies that more robust research and development activities are required in these areas to realize the opportunities of these technologies in enhancing SM capabilities of supporting decisions making in healthcare SCM.

## 7. Concluding Remarks

Over the decades a variety of organizations including healthcare began using computer modeling and simulation to enhance their operations. Simulation modeling (SM) emerged as a tool to develop specific functional and decision systems that provided flexibility, specificity, and consistency. Healthcare simulation modeling is a way to test changes in a computerized environment that will hopefully put forward ideas for improvements and subsequent implementation. The information presented from the research literature on various models was applied to support the healthcare SC decision making process. The experience and potential value of how models may be applied are a useful tool in promoting better understanding of these processes.

By offering direct feedback on suggested changes, SM allows healthcare supply chain organizations to analyse different scenarios for decision making while encouraging open communication to further understand the inner workings of a potentially complex system. Implementing SM in healthcare SCM requires the candidate organization to be well structured, integrated, and prepared to implement and use such a system. There are many strategies (approaches) for modeling healthcare SCM that can be used depending on the problem and the results the organization is trying to achieve.

As steps are being developed for the success of simulation, it should be viewed not just in the use of current and future technologies but also its application to the clinical environment. As new technologies emerge to mitigate concerns regarding implementation, potential impact, and value added for healthcare SC processes, it then becomes necessary for healthcare organizations to realize the likelihood of simulation modeling to enhance their operations and maximize the benefits.

## Figures and Tables

**Table 1:** Summary data on proposed simulation models to support decisions making in healthcare SCM.

SM	SCM scope	Decision level	Problem type and description	Modeling approach	Decision variables	Objective functions	Monetary value	Customer service initiative	Assumptions and constraints	Issues
[37]	Management-Distribution (drugs—outpatient clinics)	Planning	Optimization Manage fair and equitable scare drug distribution for outpatient clinics	Deterministic (multiobjective) Allocation heuristic solution	Dollar value of drug *k* received by clinic; 0-1 variable represents whether the clinic obtained any allocation of drug *k*	Minimize left over budget; minimize differences between allocation ratios and orders	Asset utilization	Product availability	Clinic constraints (clinics do not exceed their allocated budget); pharmaceutical firm constraints (dollar value of total disbursement does not exceed the limits in the settlement agreement); allocation constraints (dollar value of allocated drug to a clinic is ≤ the ordered amount; and meet at least the minimum order quantity by each clinic for each drug)	Complexity; scalability; generalization

[31]	Management Inventory management (drugs—Inpatient pharmacy)	Planning	Optimization Determine the optimal inventory policy for pharmaceutical drugs	Stochastic (Markov decision process)	Inventory level; expected patients' demands; volume (drug order quantities)	Minimize wastage and holding cost; maximize timely access	Cost behavior	Product availability and response time	No back-logging of demand; demands to be fulfilled at the same day even if it involves procuring the drug from different hospital;	Tie patient type to demand variability; drug availability from other facilities

[38]	Management Inventory management (all products—hospital)	Strategic and planning	Optimization Determine the optimal stock levels of overall products in hospitals	Stochastic (constraint programming)	Service level; frequency of delivery; stock-up amount	Maximize the minimum service level; maximize the average service level	Asset utilization	Product availability	Products supplied in regular (normally distributed) manner; Inventory constraint (relationship between decision variables is kept consistent); space constraint; criticality constraint (users can impose constraints to fix a product to highest level: 99%)	Demand in hospitals usually exhibits highly dynamic and uncertain pattern

[39]	Design logistic process (flow of sterile instruments from CSSD to OT)	Strategic and planning	Optimization Redesign SCM process to optimize the work process for sterilization logistics	Hybrid (dynamic programming)	Capacity; frequency of delivery; the extent of outsourcing	Minimize the total cost (transportation; OT storage; instrument usage cost)	Cost behavior	Product availability and response time	Outsourcing CSSD; the sterile net can be used only once per day; demand satisfaction	Counterbalance the increase in transportation cost; i**ncrease** OT storage capacity to handle weekly nets supplies

[10]	Design logistic process (overall SCM)	Strategic and planning and operational	Optimization Re-design the configuration of the hospital SCM; assuring efficient process and sufficient inventory level	Stochastic (graph theory); Heuristic approaches: Tabu Search (TS); and Variable Neighborhood Search (VNS)	Volume (inventory quantities at each node)	Minimize total cost of inventory (acquisition transportation; administrative; inventory carrying)	Asset utilization and cost behavior	Product availability and response time	Administrative cost is fixed; storage constraints are constant through the planning process; demand satisfaction; aggregating all items; producers supply capacity affect all potential buyers; flow persistence between SC members	Expensive computing; sudden increase in demands

[27]	Design logistic process (overall SCM)	Strategic and planning and operational	Optimization Re-design the configuration of hospital SCM assuring sufficient inventory level and manpower resources	Stochastic (optimal control theory); Heuristic approaches: Tabu Search (TS); and Variable Neighborhood Search (VNS)	Service sequence (suppliers deliver at period *t*; CU is visited at period *t*); manpower time.	Minimize total cost of inventory and human resources cost	Asset utilization and cost behavior	Product availability and response time	Time-restrictions on manpower to accomplish tasks; direct deliveries; products replenished for only suppliers' who visit the hospital; products replenished at only visited units; storage capacity; demand satisfaction	Expensive computing; details of supply work schedules; failed to provide tight schedule as optimal solution
